# Geltrex-based protocol for the differentiation of rat adipose tissue–derived mesenchymal stem cells into insulin-producing cells: in vitro and in vivo considerations

**DOI:** 10.1007/s00210-025-04902-5

**Published:** 2025-12-29

**Authors:** Hanaa H. Ahmed, Nadia S. Mahmoud, Hadeer A. Aglan

**Affiliations:** 1 Hormones Department, Medical Research and Clinical Studies Institute, National Research Centre, 33 El Buhouth St. (Former El-Tahrir St.), P.O. 12622, Dokki, Giza, Egypt; 2 Stem Cell Lab, Center of Excellence for Advanced Sciences, National Research Centre, Dokki, Giza, Egypt

**Keywords:** Adipose tissue–extracted mesenchymal stem cells, Diabetes mellitus, Geltrex-based differentiation protocol, Insulin-producing cells, Rats

## Abstract

Diabetes mellitus (DM) is a chronic illness manifested by uncontrolled glycemic levels. The present investigation was designed to explore the role of Geltrex, integrated with inducing factors, in driving the differentiation of rat adipose tissue–extracted mesenchymal stem cells (ADSCs) into efficient insulin-producing cells (IPCs) and to appraise the curative effect of the generated IPCs against type I DM rat model elicited by intraperitoneal injection of streptozotocin. ADSCs were harvested, characterized, and stimulated to differentiate into IPCs using a Geltrex matrix supplemented by a mixture of growth factors and other differentiation-inducing factors. The identity of the resultant IPCs was affirmed by measuring the expression levels of IPC-related genes and an insulin secretion assay. Then, the therapeutic efficacy of the well-characterized IPCs was assessed *via* implantation in a diabetic rat model. The Geltrex-based differentiation protocol successfully generated functional IPCs displaying upregulated levels of pancreatic endocrine genes. Moreover, the PKH-26 staining monitored the engraftment of IPCs into the pancreas of the treated rats. Additionally, IPC transplantation in diabetic rats elicited a significant enhancement in metabolic indices and motivated a significant overexpression of the pancreatic-specific genes. The therapeutic impact of the inoculated IPCs was further evidenced by the remarkable improvement in the histological architecture of the pancreatic tissue of the treated rats. The Geltrex-dependent differentiation protocol could be an optimum one for producing functional IPCs, derived from rat ADSCs, which could be an essential precept for future clinical application in the treatment of DM.

**Graphical Abstract:**

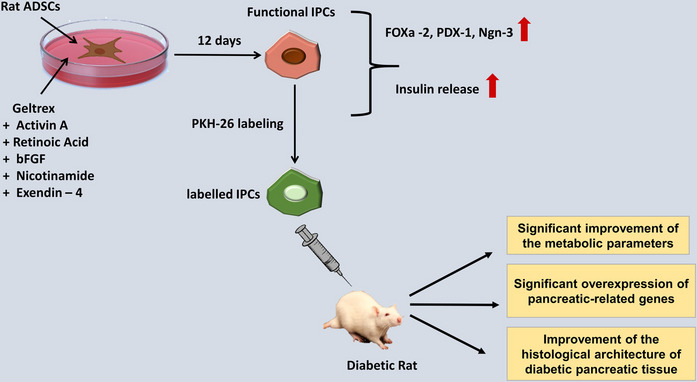

**Supplementary information:**

The online version contains supplementary material available at 10.1007/s00210-025-04902-5.

## Introduction

Diabetes mellitus (DM) is a chronic metabolic ailment defined by continuous hyperglycemia as a result of the body’s inability to produce insulin or failure to respond to insulin. DM is a global health burden that is assumed to affect 537 million individuals worldwide, and this number is anticipated to grow to 643 million by 2030 and 783 million by 2045. DM is classified into type I, which arises from autoimmune dysfunction of insulin-secreting pancreatic beta cells, and type II, which is characterized by resistance to insulin action (Hossain et al. 2024).

Type I diabetes can be managed through lifestyle changes, medical management, and regular monitoring of blood glucose levels. The most commonly available oral medications to manage elevated blood glucose levels include sulfonylureas, metformin, dipeptidyl-peptidase-4 (DPP-4) inhibitors, sodium-glucose transporter-2 (SGLT2) inhibitors, and glucagon-like peptide (GLP-1) agonists (Yameny 2024). However, current anti-diabetic medications fail to reverse the disease or prevent its complications, consequently failing to maintain perfect control of glycemic levels. Regarding type Ⅰ DM, islet transplantation can offer an effective therapy for this type of DM. However, the main hurdles facing islet transplantation are the immunosuppression problem and the limitations of donor supply. Alternatively, stem cell–derived insulin-producing cells represent a limitless supply for treating type Ⅰ DM (Ghoneim et al. 2024).

Mesenchymal stem cells (MSCs) have demonstrated promising therapeutic efficacy in diabetes, as shown by both preclinical and clinical research. In contrast to embryonic stem cells, MSCs represent an attractive source for curing type Ⅰ diabetes since they do not raise any ethical issues besides their wide availability. Moreover, MSCs can regenerate and differentiate into mesodermal lineages, such as adipogenic, chondrogenic, and osteogenic lineages. Moreover, they exhibit low immunogenicity due to the lack of major histocompatibility complex (MHC) class II on their surface. A recent meta-analysis study suggests that MSCs may have antidiabetic activity, as multiple doses of MSCs may help reduce hyperglycemia in DM individuals (Habiba et al. 2024). MSCs can exert this therapeutic benefit *via* various mechanisms; they can migrate to injury sites and aid in the repair of pancreatic islets by engrafting and differentiating into required cell types (Ezquer et al. 2008), they promote the vascularization of islet grafts, and their immunomodulatory properties also offer a potential therapeutic strategy for early-onset type Ⅰ DM. However, numerous studies have shown that MSCs are not easily integrated into target tissues, and some of the transplanted cells often die or get destroyed (Ghoneim et al. 2024). Therefore, a multitude of studies have been directed to discover new protocols for *in vitro* differentiation and propagation of β-cells to enhance their yield for pancreatic islet transplantation therapy. Stem cell differentiation into insulin-producing cells (IPCs) seems to be a promising strategy to produce a sufficient amount of functional β-cells for transplantation. Various differentiation protocols have been applied using a mixture of cytokines or signaling modulators to stimulate or constrain the key signaling pathways that orchestrate stem cell differentiation into pancreatic β-cells (Silva et al. 2022).

Despite the potential of IPC-dependent therapy for diabetes, many challenges should be addressed before it can be applied in clinical practice. One major challenge is attaining mature functional IPCs, capable of producing sufficient insulin in response to physiological cues regulating their secretion. Usually, IPCs derived from MSCs produce an insufficient amount of insulin in response to glucose. This may be attributed to the partial maturation of β cells in vitro owing to the deficit of mesenchymal-epithelial interactions that occur during pancreatic development (Pokrywczynska et al. 2013). Consequently, more research is required to establish reliable and effective protocols for generating mature functional IPCs from MSCs that closely simulate the behavior of normal pancreatic β cells. Moreover, optimizing these protocols based on the cell type is essential. Therefore, this study was designed to examine the efficacy of the differentiation protocol based on the Geltrex basement membrane matrix combined with external inducing factors to enhance the proliferation and differentiation of rat ADSCs into functional IPCs. Additionally, the anti-diabetic efficacy of the generated IPCs was evaluated in a type Ⅰ diabetic rat model.

## Material and methods

### Harvesting and propagating rat ADSCs

Adipose tissue was extracted from subcutaneous and abdominal fat tissue of 10 male rats (*Sprague Dawley*) of 200–230 g in weight (age of 8–9 weeks) under anesthesia *via* intraperitoneal injection of ketamine (75 mg/kg) and midazolam (10 mg/kg) (Valentim et al. 2013). Adipose tissue was minced and suspended in PBS (Biowest, France), followed by digestion using collagenase II (0.075%) (Serva Electrophoresis, Germany) for 1 h at 37 °C in a shaker incubator. The resultant tissue was then filtered and centrifuged at 250 × g for 10 min at 25 °C. The erythrocytes were eliminated using erythrocyte lysis buffer. The cell pellet was suspended in DMEM (Biowest, France) supplied with FBS (10%; Biowest, France) and incubated at 37 °C in a CO_2_ incubator (5%) for 24 h (Alhadlaq and Mao 2004). After that, the floating cells were eliminated by washing with PBS, while the remaining adherent cells were replenished in basal media. Once ADSCs developed a complete cell sheet, they were rinsed twice with PBS and digested by trypsin/EDTA (0.025%) (Biowest, France) for 3 min at 37 °C for cell passaging. Third-generation ADSCs were used in subsequent experiments.

### ADSC identification

Third-passage ADSCs were morphologically recognized under an inverted microscope, and further definition was performed by analyzing the MSC-specific markers (CD90 and CD105) using flow cytometry. ADSCs were incubated with antibodies against CD90 (1:100), CD105 (1:100), and CD45 (1:100) acquired from Thermo Fisher Scientific, USA, for 45 min at 4 °C in the dark, followed by scanning by a flow cytometry device (Beckman Coulter Life Sciences, USA). Isotype control rat immunoglobulin was utilized to detect background staining.

### Multipotent differentiation capacity

#### Adipogenic differentiation

ADSCs were differentiated into adipocytes by incubating in a StemPro® adipogenic differentiation medium, obtained from Gibco, for 2 weeks. Afterward, the resultant adipocytes were verified by the presence of lipid-containing vacuoles upon Oil Red O staining (Sigma, USA).

#### Chondrogenic differentiation

The differentiation of ADSCs into chondrocytes was established by suspending them in StemPro® chondrogenic differentiation medium (Gibco, Thermo Fisher Scientific, USA). Two weeks later, the differentiated cells were fixed with formaldehyde (4%) for 30 min before being stained with 1% Alcian Blue solution (Sigma, USA) for 30 min. The resultant chondrocytes were confirmed by the presence of proteoglycans stained in blue upon examination under the inverted microscope.

#### Osteogenic differentiation

The osteogenic differentiation of ADSCs was achieved by seeding in StemPro® osteogenic differentiation medium (Gibco, USA) for 3 weeks. The osteocytes were validated by 2% Alizarin Red S (Sigma, USA) staining, which indicated the calcification of the extracellular matrix.

### IPCs-differentiation of ADSCs

ADSCs were directed to differentiate into IPCs as designated by Wang et al. (2012). Briefly, ADSCs (5 × 10^5^ cells/well) were seeded in Geltrex™-coated 6-well plates (Gibco) supplied with LG-DMEM and FBS (15%) for a day. After that, the medium was substituted with HG-DMEM, supplemented with FBS (2%) and activin A (100 ng/mL, STEMCELL Technologies) for another day. The cells were then maintained in HG-DMEM provided with FBS (2%) and 1 µM retinoic acid, obtained from Sigma-Aldrich, for another 24 h. Thereafter, the cells were suspended in HG-DMEM supplemented with FBS (2%) along with basic fibroblast growth factor (10 ng/mL, STEMCELL Technologies). Three days later, the growth medium was substituted with high-glucose DMEM supplemented with FBS (2%) and nicotinamide (10 mmol/L) for 72 h. Finally, the cells were stimulated with HG-DMEM supplied with FBS (2%) and nicotinamide (10 mmol/L) in combination with exendin-4 (10 mmol/L) for another 72 h.

### Validation of the functional features of the generated IPCs

#### Quantitative analysis of IPC-related genes

RNA was extracted from the generated insulin-secreting cells using an RNeasy kit (Qiagen, Germany) according to the recommended protocol. RNA quality and concentration were estimated by NanoDrop 2000 (Thermo Fisher Scientific, Rockford, IL, USA). Then, cDNA was synthesized using a cDNA synthesis kit (Thermo Fisher Scientific, Lithuania). mRNA transcriptional levels of forkhead box protein A2 (Foxa2), pancreatic and duodenal homeobox 1 (PDX-1), and neurogenin-3 (Ngn-3) were evaluated using a DNA Technology qPCR device (Russia). PCR reactants comprised QuantiTect SYBR Green master mix (Qiagen, Germany), sense and antisense primers of the interested genes (Invitrogen, USA), cDNA, and nuclease-free water. GAPDH was utilized as an internal reference gene. Comparative mRNA transcriptional level with respect to the control was estimated using the 2^−ΔΔCt^ equation. The sequences of primers are tabulated in Table 1.

**Table 1:** Primers used in the qRT-PCR procedure

Gene	Sense (5′−3′)	Anti-sense (5′−3′)	Reference
Foxa2	TGAAGCCCGAGCACCATTAC	CCAGGGTAGTGCATGACCTGTT	Zhang et al. (2018)
PDX-1	GGTGCCAGAGTTCAGTGCTAA	CCAGTCTCGGTTCCATTCG	Wu et al. (2007)
Ngn-3	CTTCACAAGAAGTCTGAGAACACCAG	CTGCGCATAGCGGACCACAGCTTC	
GAPDH	CACCCTGTTGCTGTAGCCATATTC	GACATCAAGAAGGTGGTGAAGCAG	

#### Insulin secretion assay

Both undifferentiated and differentiated ADSCs (IPCs) were washed twice with PBS and initially incubated in low-glucose DMEM (1 g/L) for 24 h at 37 °C, followed by incubation in DMEM containing 5.5 g/L glucose (high-glucose) for another 24 h at 37 °C in a 5% CO_2_ incubator (Tariq et al. 2013). Secreted insulin levels were measured in the supernatants of cells using a rat insulin ELISA kit purchased from Wuhan Fine Biotech, China, according to the associated pamphlet. The supernatant of undifferentiated MSCs was used as a negative control.

### Cell tracking assay

The produced IPCs were stained with PKH-26 fluorescent dye (Sigma-Aldrich, USA), following the manufacturer’s protocol, before being infused into the diabetes-afflicted rats. The pancreas tissue was investigated using an inverted fluorescent microscope (Olympus, Japan) to identify and trace the PKH-26-stained cells.

### In vivo experiment

#### Rats

In the present investigation, 30 six-week-old male rats of Wistar strain (150–170 g) were obtained from a breeding house of the Animal Care Unit (National Research Centre, Giza, Egypt). The rats were subjected to ambient temperature and humidity throughout the experimental period and provided freely with water and a rodent pellet diet. Rats were acclimatized to these conditions for 2 weeks before starting the experiment. The experimental protocols were approved by the Institutional Ethical Committee of Medical Research of the National Research Centre, Giza, Egypt (approval number 19109), complying with the ARRIVE guidelines.

#### Establishment of DM type I

After an overnight fast, a single dose of streptozotocin (STZ, Sigma, USA) (50 mg/kg) was intraperitoneally injected in rats for diabetes induction (Akinlade et al. 2021). STZ was prepared with a sodium citrate solution (50 mM, pH 4.5) supplemented with NaCl (150 mM). Three days later, the fasting blood glucose level was quantified to verify the establishment of the type Ⅰ DM model using the kit provided by Chemelex S.A. (Spain). Rats displaying blood sugar levels > 300 mg/dL were enrolled in the study.

#### Rat allocation

Animals were distributed into three groups (each comprising 10 rats): (1) control group, which was intravenously injected with a single dose of saline *via* tail vein; (2) untreated DM group, which was left untreated; and (3) DM + ADSCs-derived IPCs group comprising diabetic rats infused with 5 × 10^6^ IPCs/rat (single dose) *via* tail vein. One month later, blood samples were withdrawn from the tail veins of rats under anesthesia by intraperitoneal injection of ketamine (75 mg/kg) and midazolam (10 mg/kg). Then, the serum specimens were obtained by centrifugation for 15 min at 1800 × g at 4 °C using a cooling centrifuge and frozen at − 20 °C for biochemical analyses. Afterward, the rats were euthanized, following anesthesia, by cervical displacement to excise the pancreas tissue. The excised pancreas was rinsed with ice-cold saline and separated into three portions. The first portion was weighed and homogenized in Tris–HCl (50 mM, pH 7.4) to obtain 10% homogenate (W/V) using a cell homogenizer (El-Borady et al. 2020), whereas the 2nd portion was maintained at − 80 °C for the gene expression assay. The last portion of the pancreatic tissue was preserved in 10% neutral-buffered formalin for histological procedures.

### Appraisal of the diabetes-ameliorative effect of IPCs

#### Estimation of diabetes-relevant biochemical parameters

The serum levels of insulin (INS), C-peptide (CP), along with pancreatic glucagon (GC) levels, were estimated by rat ELISA kits procured from SinGeneClon Biotech Co., Ltd (China) according to the manufacturer’s associated pamphlets.

#### Gene expression procedure for pancreatic-specific genes

RNA was isolated from the rat pancreatic tissues with the aid of the RNeasy mini kit (Qiagen, Germany). RNA purity and concentration were validated by NanoDrop 2000 (Thermo Fisher Scientific, Rockford, IL, USA). Then, cDNA samples were synthesized using a cDNA synthesis kit (Thermo Fisher Scientific, Lithuania). Gene expression patterns of pancreatic Foxa2, fibroblast growth factor (FGF-10), insulin-like growth factor I (IGF-1), and SRY-box transcription factor 17 (SOX-17) were estimated using a qPCR device of DNA-Technology (Russia). PCR contained QuantiTect SYBR Green master mix (Qiagen, Germany), nuclease-free water, sense and antisense primers of the investigated genes (Invitrogen, USA), and cDNA template. The differential mRNA transcriptional patterns relative to the control value were analyzed using the 2^−ΔΔCT^ equation after being normalized with the GAPDH gene. The primers of the investigated genes are tabulated in Table 2.

**Table 2:** Primer sequences of pancreatic-specific genes

Gene	Forward	Reverse	Reference
Foxa2	5՝-TGAAGCCCGAGCACCATTAC-3՝	5՝-CCAGGGTAGTGCATGACCTGTT-3՝	Zhang et al. (2018)
SOX-17	5՝-GGCGCCAGCCGGGACCTC-3՝	5՝-GGCCGCCCTCGGGACCAA-3՝	Wang et al. (2012)
IGF-1	5՝GCTTTTACTTCAACAAGCCCACA-3՝	5՝-TCAGCGGAGCACAGTACATC-3՝	Imberti et al. (2015)
FGF-10	5՝-TTGCTCTTCTTGGTGTCTTCC-3՝	5՝-ACCTTGCCGTTCTTTTCAATC-3՝	
GAPDH	5՝-CACCCTGTTGCTGTAGCCATATTC-3՝	5՝-GACATCAAGAAGGTGGTGAAGCAG-3՝	Wu et al. (2007)

### Histological procedure

The pancreatic tissues were dehydrated using a series of diluted ethanol after being immersed in buffered formalin (10%) for 24 h. After that, pancreatic tissues were cleared using xylene, immersed in paraffin wax, and fractionated into sections (5–6 µm) using a microtome. Subsequently, the tissue sections were deparaffinized and stained with hematoxylin and eosin for histological visualization under a light microscope (Olympus BX51, Shinjuku, Japan), following the method of Bancroft and Gamble (2008).

### Statistical study

The present findings are displayed as mean ± standard deviation (SD). Statistical analysis was performed using SPSS software (version 20). The independent sample *t*-test was used for comparison between two groups, whereas one-way analysis of variance (ANOVA) was used for multi-group comparisons, succeeded by the least significant difference to evaluate the significant difference among the studied groups. *P* value < 0.05 was deemed significant. The data were checked for normal distribution using the Shapiro–Wilk test (Supplementary Table 3).

## Results

### Verification of the ADSC-specific features

#### ADSCs morphology and cell surface profile

**Fig. 1:**
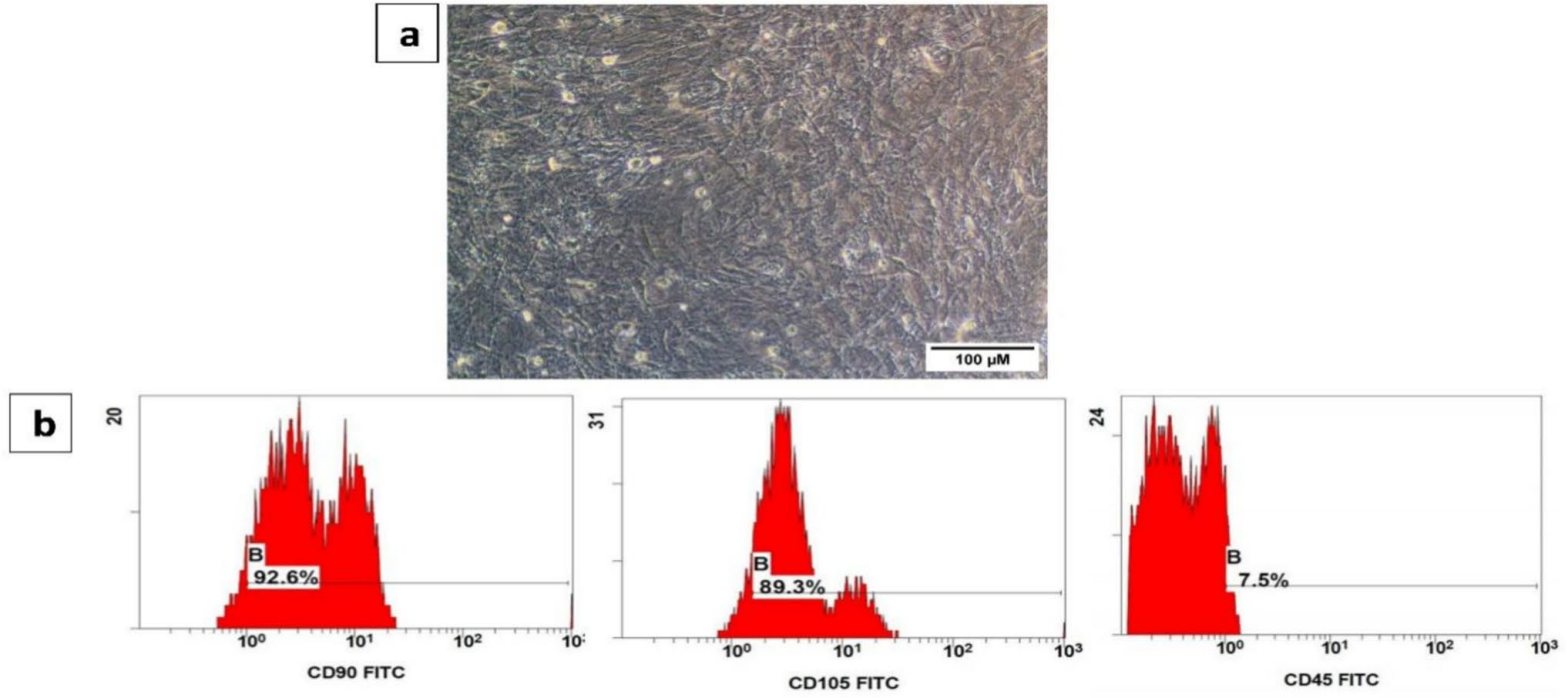
Characterization of the isolated ADSCs. **a** Photomicrograph displaying a fusiform-like appearance of ADSCs culture under an inverted microscope, **b** CD90, CD105, and CD45 immunostaining of ADSCs using flow cytometry analysis

The rat ADSCs population demonstrated adherent spindle-shaped morphology upon microscopic observation, as indicated in Fig. 1a. Moreover, these cells displayed positive immune reactions with CD 90 (92.6%) and CD 105 (89.3%) and a weak reaction for CD 45 (7.5%) upon flow cytometry analysis (Fig. 1b).

#### ADSCs tri-lineage differentiation

**Fig. 2:**
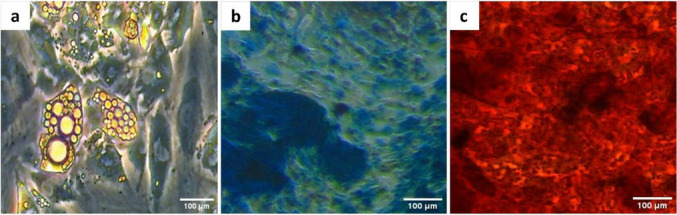
Multipotent differentiation of rat ADSCs into** a** adipocytes, **b** chondrocytes, and **c** osteoblasts

Rat ADSCs were effectively converted into adipocytes, chondrocytes, and osteoblasts. The differentiation of a small subset of ADSCs into adipocytes was validated by Oil Red O staining (Fig. 2a), whereas Alcian Blue staining confirmed the synthesis of sulfated proteoglycan by chondrocytes (Fig. 2b). Calcified nodule deposition in the extracellular matrix of osteocytes was verified by Alizarin red staining (Fig. 2c).

### Functional features of ADSC-derived IPCs

**Fig. 3:**
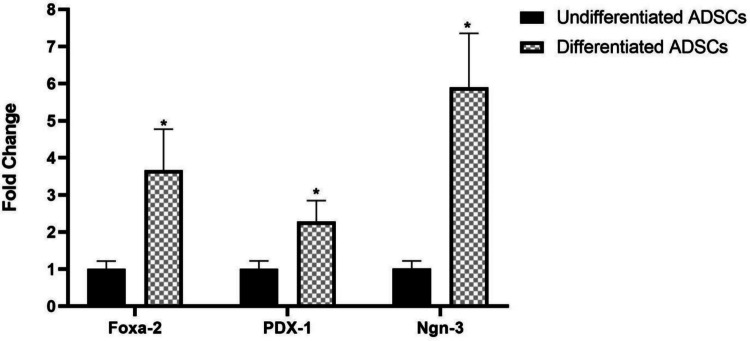
IPC-specific gene expression patterns of IPCs derived from ADSCs. Data are denoted as mean ± SD of 3 technical replicates (*n* = 3)..*Significant variation at *P* < 0.05 as compared to the negative control (undifferentiated ADSCs)

We profiled the functional features of IPCs derived from ADSCs differentiation for 12 days by analyzing the IPC-specific genes using qRT-PCR. The generated IPCs exhibited a significant (*P* < 0.05) upregulated level of Foxa-2, PDX-1, and Ngn-3 genes *versus* the undifferentiated ADSCs (Fig. 3).

**Fig. 4:**
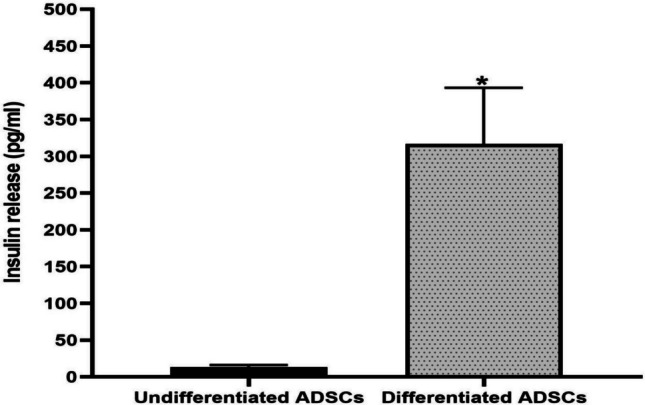
Insulin secreted from IPCs generated from ADSCs. Data are expressed as mean ± SD of 3 technical replicates (*n* = 3). *Significant variation at *P* < 0.05 relative to the negative control (supernatant of undifferentiated ADSCs)

Also, the *in vitro* insulin secretion from IPCs differentiated from ADSCs responding to high glucose concentration was determined to validate the functional entity of the generated IPCs. IPCs differentiated from ADSCs released a significantly (*P* < 0.05) elevated insulin level responding to elevated glucose levels (316.7 ± 76.4 pg/mL), contrary to the undifferentiated ADSCs (13.3 ± 2.1 pg/mL) (Fig. 4).

### Engraftment of implanted IPCs into the pancreatic tissues

**Fig. 5:**
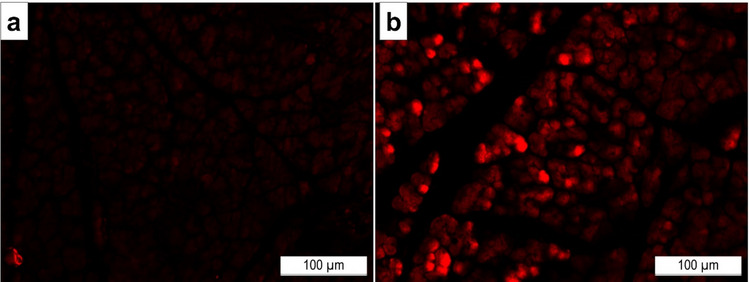
Engraftment of the transplanted IPCs to the pancreatic tissue of the IPCs-treated rats.** a** Fluorescent photography of the pancreatic tissue of an untreated DM rat. **b** Fluorescent photography of the pancreatic tissue of DM rats treated with labeled IPCs

Microscopic examination of pancreatic tissue obtained from diabetic rats treated with IPCs (generated from ADSCs differentiation) yielded many PKH-26-stained cells, declaring the successful engraftment of infused IPCs to the diabetic pancreas of the treated rats (Fig. 5), as compared to the untreated diabetic group showing no PKH-labeled cells (no red fluorescence) and served as a valid negative control.

### Biochemical outcomes

**Fig. 6:**
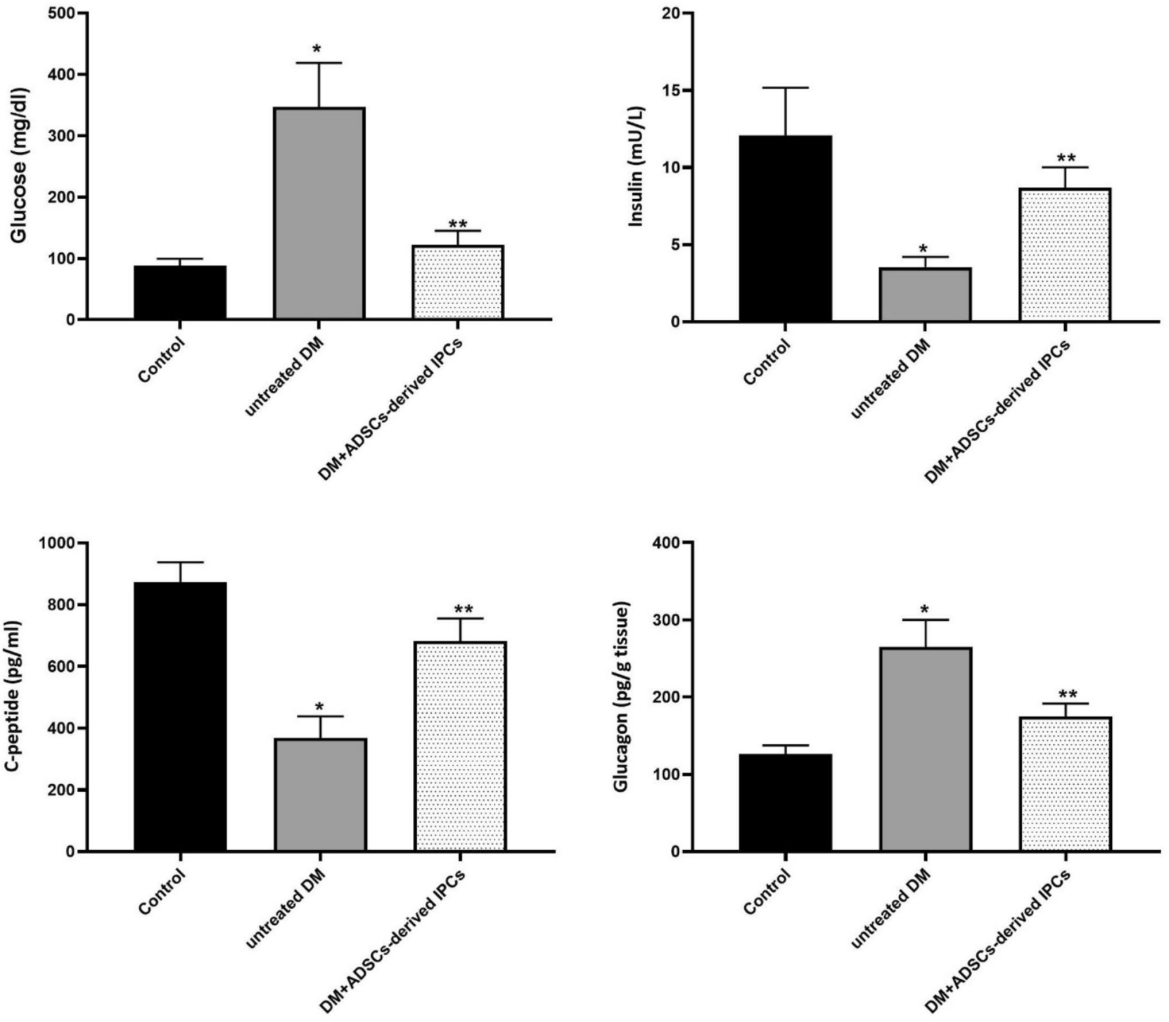
Impact of IPCs infusion in diabetic rats on serum glucose, insulin, and C-peptide, along with pancreatic glucagon levels. Data are denoted as means ± SD (*n* = 8 per group). *Significant variation at *P* < 0.05 in contrast with the control group, **significant variation at *P* < 0.05 in contrast with the untreated diabetic group

The influence of treatment with ADSC-derived IPCs on serum glucose, insulin, and C-peptide, along with pancreatic glucagon levels in the diabetic rats is illustrated in Fig. 6. The untreated diabetic group revealed a significant rise (*P* < 0.05) in serum glucose (347.1 ± 71.5 mg/dL) and pancreatic glucagon levels (265 ± 34.9 pg/g tissue) *versus* the corresponding control values (88.3 ± 11.3 mg/dL and 126 ± 11.6 pg/g tissue), respectively. Moreover, this group brought about a significant (*P* < 0.05) decline in serum insulin (3.5 ± 0.7 mU/L) and C-peptide levels (368.1 ± 69.9 pg/mL), contrary to the control group values (12.1 ± 3.1 mU/L and 872.5 ± 64.9 pg/mL), respectively. On the other side, the implantation of the generated IPCs in the diabetes-induced rats provoked a significant diminution (*P* < 0.05) in serum glucose (122.3 ± 22.6 mg/dL) and pancreatic glucagon levels (175 ± 16.7 pg/g tissue), accompanied by a significant (*P* < 0.05) elevation in serum insulin (8.7 ± 1.3 mU/L) and C-peptide levels (682.5 ± 62.5 pg/mL) in contrast with the untreated diabetic rats.

### Gene expression findings

**Fig. 7:**
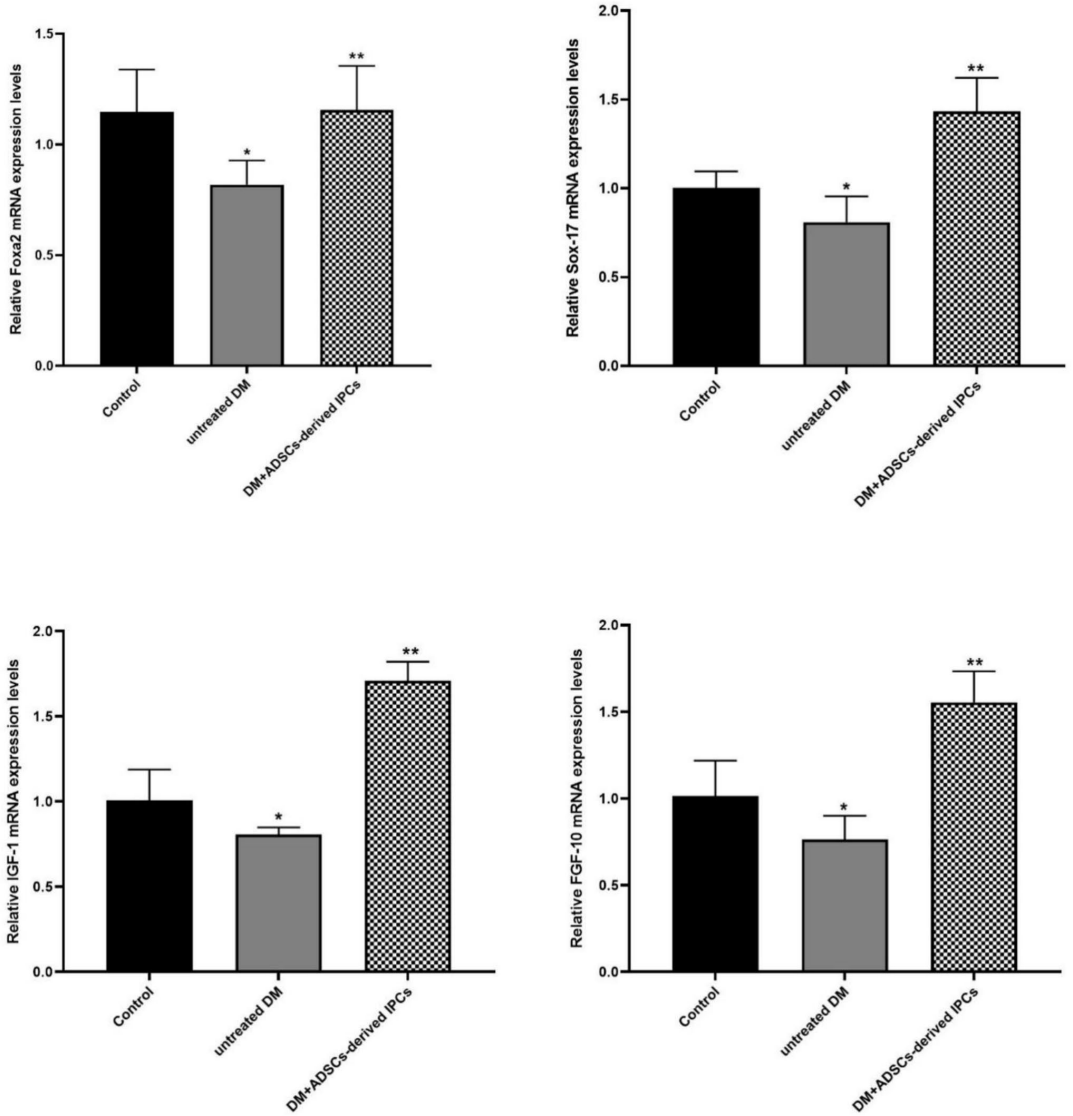
The influence of IPCs implantation on the mRNA transcriptional levels of Foxa-2, Sox-17, IGF-1, and FGF-10 in the DM group. Data are represented as means ± SD (*n* = 6 per group). *Significant variation at *P* < 0.05 *versus* the control group, **significant variation at *P* < 0.05 *versus* the untreated DM group

The impact of the treatment with ADSCs-derived IPCs on pancreatic Foxa2, IGF-1, FGF-10, and SOX-17 transcriptional patterns in diabetes-afflicted rats is shown in Fig. 7. The untreated DM rats elicited a significant down-expression (*P* < 0.05) in the pancreatic Foxa2, IGF-1, FGF-10, and SOX-17 gene levels relative to the control counterparts. On the other hand, the IPC implantation in diabetic rats evoked a significant over-expression (*P*  <0.05) in Foxa2, IGF-1, FGF-10, and SOX-17 gene levels, as compared to the untreated diabetic rats.

### Histological observations

**Fig. 8:**
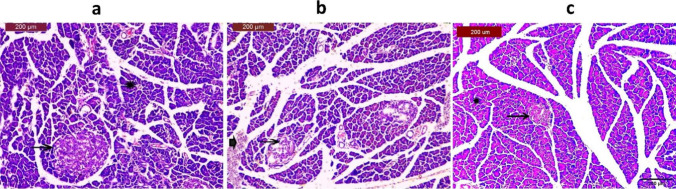
Histological examination of pancreatic tissue sections obtained from** a** control group demonstrated normal pancreatic parenchyma (*) and regular structure of pancreatic islets (→), **b** untreated diabetic rat displayed degeneration of pancreatic islets (→), associated with a hemorrhage into extended interlobular septa (➨), and **c** diabetic rat implanted with insulin-producing cells showing normal pancreatic parenchyma (*) and almost normal features of Langerhans islets (→)

The pancreatic tissue section of normal control rat exhibited normal pancreatic parenchyma and pancreatic islets (Fig. 8a), while the pancreatic tissue section of the untreated diabetic rat manifested disintegration of pancreatic islets associated with hemorrhage into extended interlobular septa (Fig. 8b). Histopathological examination of pancreas tissue section obtained from diabetes rat implanted with IPCs, derived from rat ADSCs, indicated more or less normal pancreatic parenchyma with almost normal appearance of Langerhans islets (Fig. 8c).

## Discussion

Among several stem cell types, ADSCs are identified as the most favorable MSCs owing to their wide availability and easy derivation from adipose tissue through a less invasive isolation method compared to the bone marrow type. Moreover, the multi-potency of ADSCs enables their differentiation into numerous cell types, ensuring their eligibility for different regenerative implementations. Their renewable characteristics are interposed by the release of numerous cytokines and growth-inducing factors, which promote tissue healing and angiogenesis. Furthermore, immunomodulatory advantages and null immunogenicity elicited by ADSCs make them eligible for allogeneic transplantation with a minimal susceptibility for graft rejection. They are exceptionally proliferative cells that can be *in vitro* cultivated, sustaining genetic integrity for a long time, and can be employed in different tissue repair procedures without causing any ethical concerns because they are derived from adult tissue (Papadopoulos et al. 2024).

The current study affirmed the identity of the isolated rat ADSCs as evidenced by the spindle shape appearance upon microscopic investigation and the positive immunostaining for CD90, CD105, and mild immunostaining for the hematopoietic lineage marker (CD45). Moreover, the mesodermal differentiation capacity of ADSCs towards adipocytes, osteocytes, and chondrocytes was proved by the lineage-specific staining (Oil Red O, Alizarin Red, and Alcian Blue staining, respectively). These results agree with those of the published article of Sayed et al. (2024) and Banu et al. (2025). Although we confirmed the identity of the isolated ADSCs by analyzing the recommended ISCT markers (CD90 and CD105) as positive MSC surface markers and CD45 as a negative marker, assessing the full ISCT panel of positive and negative surface markers would provide a more comprehensive immunophenotypic characterization of the MSCs, and this will be considered in future studies. In our study, only a small proportion of ADSCs exhibited strong lineage-specific staining, especially in the adipogenic differentiation assay. This could be attributed to several factors, including the use of ready-to-use differentiation kits, which can sometimes yield modest differentiation efficiency, and the possibility that the differentiation period was not long enough for complete phenotypic maturation. The previous study of Banu et al. (2025) confirmed the trilineage potential of ADSCs using the same differentiation kits (StemPro differentiation media, Gibco) used in our study and yielded a modest differentiation capacity upon culturing the ADSCs for 24 days in these ready-to-use differentiation media. However, in our study, we cultured ADSCs in these media for only 2 weeks for the adipogenic and chondrogenic differentiation assay; therefore, the short differentiation period may be the reason that not all ADSCs were fully differentiated and positively stained.

The isolated ADSCs were successfully induced to differentiate into IPCs using a differentiation protocol based on Geltrex basement membrane matrix alongside other differentiation-inducing factors such as activin A, bFGF, retinoic acid, nicotinamide, and exendin-4 for 12 days. This was verified by the upregulated expression levels of the pancreatic-specific genes (Foxa2, Ngn-3, and PDX-1) when compared with the non-differentiated ADSCs. Furthermore, our data revealed that the generated IPCs significantly respond to glucose stimulation by secreting insulin, as documented by the insulin release assay, which further confirmed the functional maturation of the pancreatic progenitors into IPCs. These attained findings were corroborated by those of Wada et al. (2019) and Dang Le et al. (2022).

In the current work, we primarily focused on measuring insulin secretion under high-glucose conditions to evaluate the glucose-responsiveness of the induced IPCs. It has been reported that insulin secretion is basal under low glucose conditions, as pancreatic β-cells remain in a resting state. Low glucose does not provide sufficient metabolic stimulation to raise the ATP/ADP ratio, close ATP-sensitive K^+^ channels in the β-cell membrane, or trigger calcium influx, all of which are essential for insulin exocytosis. Thus, insulin release at low glucose is minimal and mainly reflects a basal leakage rather than true secretion (Henquin 2021). Therefore, insulin secretion was only assessed in response to high glucose.

Many differentiation protocols have been designed to mimic the normal pancreatic development through utilizing extracellular matrix (ECM), cytokines, and growth factors that act synergistically to activate the main signaling pathways implicated in the pancreatic differentiation process, including Wnt, Nodal/Activin A, FGF, retinoid, and Notch. Consequently, combining these factors simultaneously, with certain concentrations and timing, ensures the successful derivation of functional IPCs. The differentiation of MSCs into IPCs mostly entails three differentiation stages, including the formation of definitive endoderm (DE), followed by pancreatic endocrine progenitors and, eventually, maturation into β-cells. Relative gene expression analysis during this process is crucial to validate each *in vitro* differentiation stage (Silva et al. 2022). A multitude of evidence suggests that ECM supports stem cell survival and regulates their differentiation by sustaining its structural, biochemical, and biomechanical characteristics (Aly et al. 2022). It was indicated that the possible explanation for the failure of *in vitro* β-cell maturation could be attributed to the absence of extracellular environment interactions (Kumar et al. 2003). Therefore, using ECM may provide a suitable environment similar to that of the *in vivo* microenvironment necessary for the *in vitro* pancreatic differentiation of MSCs. Geltrex is a synthetic ECM that is mainly composed of key proteins, including laminin, collagen IV, entactin, and heparan sulfate proteoglycans. ECM not only provides structural support but also creates a microenvironment for cells that influences their behavior and fate. ECM, such as laminin, induces the MSC differentiation into IPCs by activating the expression of key pancreatic genes, including Foxa2, Sox17, PDX-1, and Ngn3, essential for regulating insulin expression (Qu et al. 2014). It acts by binding to αvβ3 integrin on the MSC surface, triggering intracellular signaling pathways including the extracellular-signal-regulated kinase (ERK) pathway (Peng et al. 2017). ECM proteins such as fibronectin and laminin have been shown to enhance the differentiation of MSCs into IPCs by activating Akt and ERK pathways, involved in cell proliferation and cell cycle progression (Lin et al. 2010). Moreover, Geltrex has been reported to stimulate the formation of efficient DE and act as a support to guide the differentiation of stem cells towards pancreatic lineage by simulating the *in vivo* pancreatic microenvironment required for proper cell attachment, survival, and lineage-specific signaling (Massumi et al. 2016). Hence, we applied a protocol based on Geltrex as a support for all stages of the differentiation process. Also, activin A, along with retinoic acid, bFGF, nicotinamide, and exendin-4, was utilized along with a high glucose medium supplied with low serum during the differentiation protocol steps. Activin A, belonging to the transforming growth factor β (TGF-β) family, is crucial to initiate the early pancreatic development. It can induce the differentiation of MSCs towards DE lineage, the germ layer from which the pancreas, liver, and gut arise, by mimicking the nodal signaling pathway and activating the expression of endodermal markers such as Foxa-2, Sox-17, and chemokine (C-X-C motif) receptor 4 (CXCR4) (Silva et al. 2022). Nodal activation mediates FGF, BMP, and Wnt pathways, thus motivating gastrulation (Qadir et al. 2020). However, activin A has been reported to catalyze neuronal differentiation (Rodríguez-Martínez et al. 2012). Thus, the timing and concentration of activin A are critical for efficient endodermal differentiation. Retinoic acid was reported to stimulate the generation of pancreatic progenitors and guide their differentiation towards insulin-producing β cells. Retinoic acid induces PDX-1 gene expression, a crucial transcription factor in the early development of pancreatic progenitors (Öström et al. 2008). bFGF also maintains PDX-1 expression in the endoderm and triggers β-cell differentiation (Silva et al. 2022). Nicotinamide, a poly(ADP-ribose) synthetase inhibitor, has been reported to stimulate the differentiation of pancreatic β-cells and to protect islet cells against toxic damage, owing to its antioxidant activity (Silva et al. 2022). Zhang et al. (2021) showed that nicotinamide provokes the pancreatic differentiation of human embryonic stem cells towards pancreatic precursor cells *via* the inhibition of casein kinase 1 (CK1) and Rho-associated protein kinase (ROCK), which in turn resulted in significant elevation of NKX6.1 and PDX1 gene expression levels. Also, it has been stated that nicotinamide maintains the expression of PDX-1 through the later stages of differentiation and induces β-cell development. Foxa2 is known to regulate the transcriptional expression of the PDX-1 gene in the pancreas (Cho et al. 2008). PDX-1 expression is essential for activating the transcriptional expression of the insulin gene (Ferber et al. 2000). The co-expression of C-peptide, PDX-1, homeobox protein NKX6.1 (NKX6.1), and V-maf musculoaponeurotic fibrosarcoma oncogene homolog A (MAFA) is the hallmark of β-cell maturation (Pokrywczynska et al. 2013). Hence, PDX-1 sustained expression is crucial for functional β-cell maturation. And that is why nicotinamide addition to the differentiation medium has a substantial effect on late endocrine differentiation in the present study. Exendin-4, the agonist of glucagon-like peptide-1 (GLP-1) receptor, induces β-cell proliferation and enhances the formation of β-cells from ductal progenitor cells. Exendin-4 has been recommended to drive Wharton’s jelly mesenchymal stem cell differentiation into IPCs *via* stimulation of early β-cell-specific markers such as PDX-1 and NK2 homeobox 2 (NKX2.2) (Kassem et al. 2016). Additionally, exendin-4 was reported to stimulate the differentiation of rat ADSCs into IPCs, as evidenced by the upregulated expression of pancreatic islet-related markers (PDX-1, insulin, and Glut-2) and enhancing the insulin release from IPCs (Khorsandi et al. 2016). Also, it has been stated that exendin-4 can activate PDX-1 *via* stimulating various signaling pathways, including the phosphatidylinositol-3-kinase (PI3K), hedgehog, and MAPK/ERK pathways (Sawangmake et al. 2014). Moreover, the addition of exendin-4 to the differentiation medium is essential for the expression of endocrine precursor-specific markers, including Ngn-3, paired box 4 (PAX-4), and NKX2.2. Endocrine cell specification is initiated by suppressing the Notch signal pathway and activating the pro-endocrine gene expression, Ngn-3, in some pancreatic epithelial cells (Wilson et al. 2003). Ngn-3 motivates the expression of many downstream genes, including NKX2.2, neuronal differentiation 1 (Neurod1), NKX6.1, PAX-4, paired box 6 (PAX-6), and insulin gene enhancer protein (ISL-1), which regulate the differentiation of endocrine cells. Hence, Ngn-3 expression drives the differentiation of pancreatic progenitor cells into endocrine lineages (Pokrywczynska et al. 2013).

While ADSCs are of mesodermal origin, growing evidence shows that MSCs can differentiate into cell types originating from different germ layers under specific microenvironmental cues. In our study, this process is best described as transdifferentiation, where ADSCs acquire pancreatic β-like characteristics without reverting to a dedifferentiated or pluripotent state. This was confirmed by the upregulation of key pancreatic genes (FOXa2, PDX-1, and Ngn-3) in the differentiated ADSCs (IPCs), indicating the successful transdifferentiation of ADSCs into functional IPCs following the same pathway of pancreatic development. Similar mesoderm-to-endoderm lineage transdifferentiation has been reported in previous studies where MSCs were successfully differentiated into pancreatic β-like cells under defined culture conditions, acquiring endodermal pancreatic lineage markers (Tayaramma et al. 2006; Chandra et al. 2011).

In our study, STZ administration effectively induced diabetes in rats, with those displaying blood glucose levels above 300 mg/dL classified as diabetic. However, not all rats developed hyperglycemia following STZ injection. Variability in response to STZ is well-documented and was reported by the study of Romanovsky et al. (2010), who revealed that 72 h after STZ induction, rats exhibited divergent glycemic responses; some developed hyperglycemia while others remained normoglycemic. In this study, rats had free access to food prior to STZ induction, and the amount and timing of food consumption during that period were not monitored. Moreover, the size of the pancreas, the primary target of STZ, does not correlate linearly with body weight. Consequently, the same dose of STZ (per kg body weight) could result in greater diabetogenic effect in larger animals. This could be attributed to the variability observed in diabetes induction among rats that received the same dose of STZ (50 mg/kg).

The therapeutic impact of IPCs transplantation, generated from *in vitro* pancreatic differentiation of ADSCs, in STZ-induced type Ⅰ diabetic rats was evaluated. The current findings indicate the successful engraftment of the infused IPCs into the diabetic pancreas of treated rats, as manifested by the recognition of the PKH-26-labeled IPCs in the pancreas tissues of IPC-injected rats, as compared to the pancreas tissue of the untreated diabetic rats. This result is greatly supported by the study of Park et al. (2022). The figure displaying the engraftment of PKH-stained IPCs into diabetic pancreases of rats does not include an image of the normal control group, as this group did not receive PKH-labeled IPCs, and thus, no red fluorescence was expected. However, the untreated diabetic group, which also did not receive PKH-labeled IPCs, was included in the figure to serve as a negative control, since it provides a more appropriate comparison to the IPCs-transplanted diabetic group, as both share the same diabetic background and tissue characteristics. This comparison was essential to confirm that the red fluorescence observed in the IPCs-transplanted group specifically originates from the engraftment of the PKH-labeled IPCs and not endogenous factors or autofluorescence within the diabetic pancreatic tissue. However, including an image for the normal control group would have further strengthened the baseline comparison and confirmed the specificity of the results. This will be considered in future work.

Glucagon is a hormone synthesized by the pancreatic α cells of Langerhans islets that maintains glucose homeostasis by promoting the production of hepatic glucose. Thus, the optimal blood glucose level is largely dependent on the balanced action of insulin and glucagon. Hence, glucagon serves as a glucose-mobilizing hormone, whereas insulin is a glucose-depositing hormone (Röder et al. 2016). C-peptide is recognized as a useful indicator of pancreatic β-cell function. It is produced by pancreatic β-cells as a byproduct of the enzymatic cleavage of pro-insulin to insulin (Palmer et al. 2004). The current investigation demonstrated that fasting blood glucose levels significantly increased in the untreated diabetic rats *versus* the control group, indicating hyperglycemia, and confirming the successful establishment of the diabetic model. Moreover, this group revealed a significant elevation in pancreatic glucagon accompanied by a significant diminution in serum levels of insulin and C-peptide as compared to the control group. These findings are in coherence with those of Park et al. (2022). On the contrary, IPC-infused rats demonstrated a significant depletion in fasting serum level of glucose and pancreatic glucagon in concomitant with significantly increased serum levels of C-peptide and insulin, as compared to untreated diabetic rats. These results denote the capability of the transplanted IPCs to reduce hyperglycemia and secrete functional insulin. The observed decline in blood glucose levels is registered in the study of Yu et al. (2015). Moreover, the modulation of other metabolic parameters observed following IPCs transplantation coincides with the studies of Hsiao et al. (2020), Park et al. (2022), and Nour Eldeen et al. (2024). Additionally, IPCs transplantation in the diabetic rats elicited a significant over-expression of pancreatic-specific genes, including Foxa2, IGF-1, FGF-10, and Sox17, contrary to the untreated DM rats. The upregulated expression levels of those pancreatic genes elucidate the successful pancreatic β-cell regeneration following IPCs transplantation. Furthermore, histological investigation of pancreatic tissue obtained from IPC-treated rats demonstrated a remarkable amelioration of pancreatic parenchyma and restoration of the normal appearance of Langerhans islets, indicating the regenerating potential of transplanted IPCs that successfully restore the cellular function of the pancreas and reverse the hyperglycemic status of the diabetic rats. The observed improvement in the histological architecture of the diabetic pancreatic tissue aligns with the study by Wang et al. (2018).

## Conclusion

Collectively, the findings of the current attempt revealed the success of the selected differentiation protocol based on Geltrex and other differentiation-inducing factors in driving the differentiation of rat ADSCs into mature functional IPCs capable of secreting a considerable amount of insulin in response to elevated glucose levels and able to engraft to the pancreatic tissue upon implantation into the diabetic rats. Additionally, by repairing damaged pancreatic β-cells, these implants can potentially restore normal glucose regulation, insulin production, and the metabolic indices in type Ⅰ diabetic rats. Consequently, the present preclinical approach offers a promising opportunity based on IPCs implantation for the management of type Ⅰ diabetes mellitus, as it provides a sustainable solution, reducing reliance on exogenous insulin administration. This advancement could be useful for application in clinical settings in the future.

## Limitations of the study

Despite the promising findings of the present study, some limitations must be acknowledged. One limitation of this study is that biochemical markers were evaluated only at a single time point (at the end of the experiment). Time-course analysis of biochemical markers would offer detailed insights into the progression of graft function and metabolic recovery. Moreover, evaluating the biodistribution of IPCs in non-target organs and quantifying the engrafted cells following transplantation would provide a complete understanding of cell fate. Additionally, assessing the reversibility of IPC response (insulin secretion) from high to low glucose within the same assay would strengthen the assessment of IPC responsiveness.

## Supplementary information

Below is the link to the electronic supplementary material.

ESM 1 (DOCX 67.0 KB)

## Data Availability

The datasets used and/or analyzed during the current study are available from the corresponding author on reasonable request.
