# Supplementary material for: Geltrex-based protocol for the differentiation of rat adipose tissue–derived mesenchymal stem cells into insulin-producing cells: in vitro and in vivo considerations
Source: Naunyn Schmiedebergs Arch Pharmacol. 2025 Dec 29;399(6):8429–42. doi: 10.1007/s00210-025-04902-5 (PMC13086821; doi:10.1007/s00210-025-04902-5)
Supplement: Supplementary file 1 — (DOCX 67.0 KB) [file 210_2025_4902_MOESM1_ESM.doc]

**Supplementary Table 3** Normality test of data (Shapiro-Wilk Test) across different groups for all variables in *in vitro* and *in vivo* study

| **Variables** | **Groups** | **Mean ± S.D.** | **Normality test (Shapiro-Wilk Test)** | |
| --- | --- | --- | --- | --- |
|  |  |  | **Statistics** | **P-value** |
| **Foxa-2 fold change** | Undifferentiated ADSCs | 1.01 ± 0.20 | 0.981 | 0.738 |
|  | Differentiated ADSCs | 3.66 ± 1.1 | 0.999 | 0.952 |
| **PDX-1 fold change** | Undifferentiated ADSCs | 1.01 ± 0.14 | 0.979 | 0.722 |
|  | Differentiated ADSCs | 2.28 ± 0.56 | 0.978 | 0.716 |
| **Ngn-3 fold change** | Undifferentiated ADSCs | 1.01 ± 0.21 | 0.996 | 0.886 |
|  | Differentiated ADSCs | 5.90 ± 1.45 | 0.978 | 0.716 |
| **Insulin release (pg/ml)** | Undifferentiated ADSCs | 13.3 ± 2.1 | 0.923 | 0.463 |
|  | Differentiated ADSCs | 316.7 ± 76.4 | 0.964 | 0.637 |
| **Glucose (mg/dl)** | Control | 88.3 ± 11.3 | 0.889 | 0.230 |
|  | Untreated DM | 347.1 ± 71.5 | 0.908 | 0.342 |
|  | DM + ADSCs-derived IPCs | 122.3 ± 22.6 | 0.909 | 0.345 |
| **Insulin (mU/L)** | Control | 12.1 ± 3.1 | 0.923 | 0.459 |
|  | Untreated DM | 3.5 ± 0.7 | 0.967 | 0.877 |
|  | DM + ADSCs-derived IPCs | 8.7 ± 1.3 | 0.952 | 0.735 |
| **C-peptide (pg/ml)** | Control | 872.5 ± 64.9 | 0.979 | 0.956 |
|  | Untreated DM | 368.1 ± 69.9 | 0.905 | 0.319 |
|  | DM + ADSCs-derived IPCs | 682.5 ± 62.5 | 0.851 | 0.097 |
| **Glucagon (pg/ g tissue)** | Control | 126 ± 11.6 | 0.894 | 0.252 |
|  | Untreated DM | 265 ± 34.9 | 0.886 | 0.213 |
|  | DM + ADSCs-derived IPCs | 175 ± 16.7 | 0.841 | 0.076 |
| **Foxa2 mRNA expression** | Control | 1.148 ± 0.15 | 0.905 | 0.403 |
|  | Untreated DM | 0.813 ± 0.15 | 0.849 | 0.155 |
|  | DM + ADSCs-derived IPCs | 1.156 ± 0.17 | 0.852 | 0.165 |
| **IGF-1** **mRNA expression** | Control | 0.99 ± 0.16 | 0.812 | 0.076 |
|  | Untreated DM | 0.81 ± 0.04 | 0.863 | 0.201 |
|  | DM + ADSCs-derived IPCs | 1.71 ± 0.10 | 0.853 | 0.167 |
| **FGF-10** **mRNA expression** | Control | 1.03 ± 0.16 | 0.896 | 0.351 |
|  | Untreated DM | 0.73 ± 0.13 | 0.827 | 0.101 |
|  | DM + ADSCs-derived IPCs | 1.52 ± 0.14 | 0.895 | 0.343 |
| **SOX-17** **mRNA expression** | Control | 0.98 ± 0.068 | 0.823 | 0.093 |
|  | Untreated DM | 0.80 ± 0.13 | 0.831 | 0.110 |
|  | DM + ADSCs-derived IPCs | 1.38 ± 0.13 | 0.914 | 0.463 |
